# Dislodgment and embolization of an atrial leadless pacemaker

**DOI:** 10.1016/j.hrcr.2025.09.009

**Published:** 2025-09-19

**Authors:** Samuel Reincke, Julius Nikorowitsch, Roland Richard Tilz

**Affiliations:** 1 Department of Rhythmology, University Heart Center Lübeck, University Hospital Schleswig-Holstein, Lübeck, Germany; 2 Deutsches Zentrum für Herz-Kreislauf-Forschung (DZHK), Partner Site Hamburg/Kiel/Lübeck, Lübeck, Germany

**Keywords:** Leadless pacing, Aveir DR, Aveir AR, LP, Device dislodgment

**Key Teaching Points**

• Atrial leadless pacemakers may embolize to the left renal vein within 24 hours of implantation.
• Immediate transvenous retraction and reimplantation of the same atrial leadless pacemaker is feasible.
• High body mass index, tricuspid regurgitation, atrial fibrosis, and increased right filling pressures may increase the risk of dislodgement.

## Introduction

The Aveir DR leadless pacemaker system (Abbott) is the first leadless dual-chamber pacemaker available on the market.^1^ Owing to the communication of 2 leadless pacemakers, it allows atrioventricular (AV) synchronous pacing with high reliability.^2^ Only sparse data are available concerning the dislodgment of atrial leadless pacemakers. 2 case reports describe the dislodgment of an atrial Aveir to the left ventricular outflow tract and to the femoral vein.^3^^,^^4^ However, to the best of our knowledge, this is the first case report of a dislodgement and embolization of an Aveir AR pacemaker to the left renal vein in the setting of a severe tricuspid regurgitation.

## Case report

A 72-year-old male patient presented with dyspnea. The patient had undergone a replacement of the aortic valve (mechanical), pulmonary valve (homograft), and ascending aorta 19 years before, owing to aortic stenosis and an aortic aneurysm. During this surgery, the right atrial appendage was not ligated. An incisional right atrial tachycardia (posterior wall) and atrial flutter were treated with catheter ablation 2 years before the current presentation. Electrophysiological workup revealed during rest (1) an AV block type I with a PR time of 400 ms, (2) right bundle branch block, (3) atypical left anterior fascicular block and during bicycle ergometry, (4) a chronotropic incompetence (maximum frequency 72/min), and (5) an increase of the PR interval and intermittent second-degree type I AV block. Echocardiographic assessment showed several valve issues: a severe tricuspid regurgitation and a severe regurgitation and moderate stenosis of the homograft in pulmonary position. The right ventricle was dilated (right ventricular end-diastolic diameter 52 mm) and showed a reduced function (tricuspid annular plane systolic excursion 13 mm) with increased pressures (estimated systolic pulmonary artery pressure 38 mm Hg). The right atrium was dilated (right atrial volume index 64 mL/m^2^), and the left ventricular ejection function was mildly reduced (45%). Other comorbidities included sleep apnea with the use of continuous positive airway pressure therapy and class 3 obesity with a body mass index (BMI) of 40 kg/m^2^.

**Figure 1:**
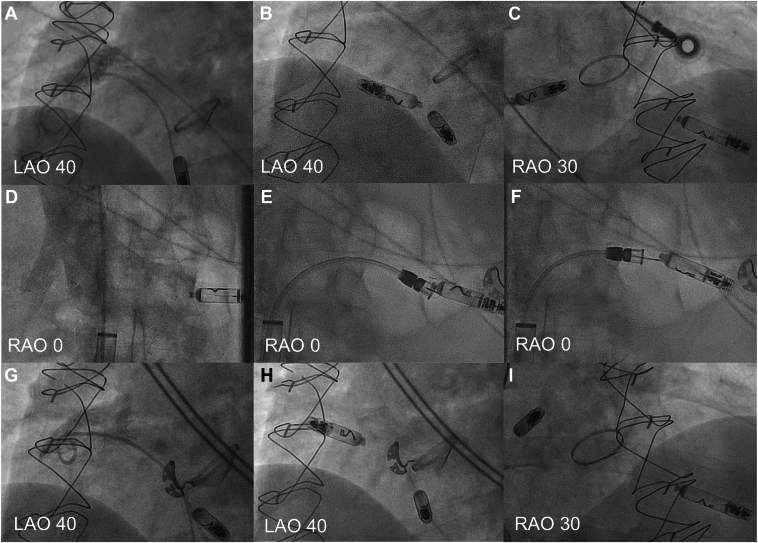
**A:** Angiographic images of the initial implantation procedure showing the right atrial appendage (RAA) with contrast and (**B**) the Aveir devices in LAO and (**C**) RAO views. Angiographic images showing the atrial leadless pacemaker in the (**D**) left renal vein, (**E**) snaring and (**F**) successful retraction of the device, (**G**) RAA in LAO, and (**H**) successful reimplantation at the base of the right atrial appendage in LAO and (**I**) RAO. LAO = left anterior oblique; RAO = right anterior oblique.

**Figure 2:**
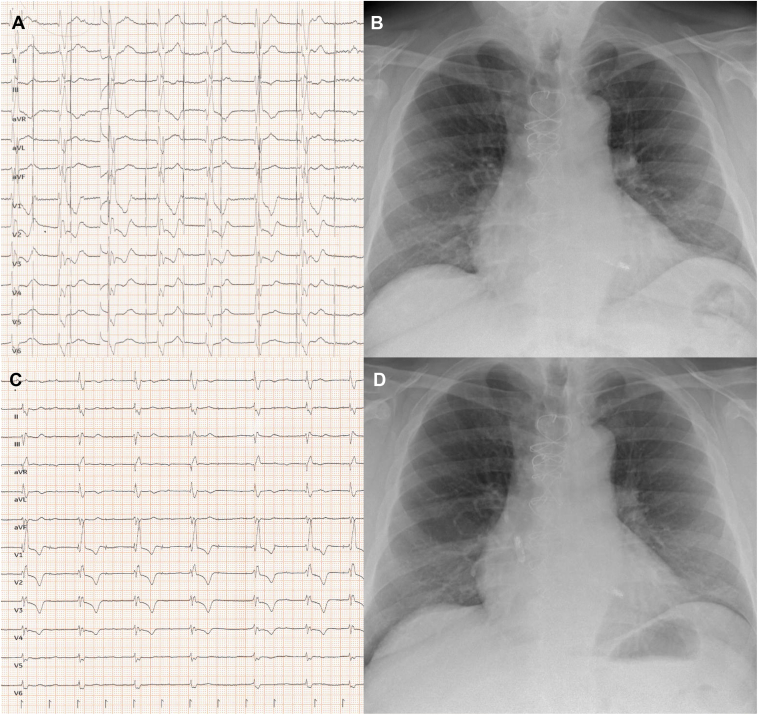
**A:** ECG showing atrial exit block after initial implantation. **B:** Chest radiograph after the implantation showing the embolization of the atrial leadless pacemaker. **C:** ECG during threshold testing in AAI after replacement of the leadless pacemaker. Note the loss of capture visible in I and II after the fourth QRS complex. **D:** Chest radiograph after successful repositioning of the atrial leadless pacemaker. ECG = electrocardiogram.

Potential worsening of the tricuspid valve regurgitation through transvenous lead implantation and the increasing mechanical dyssynchrony through right ventricular pacing were discussed. We decided to implant a dual-chamber leadless pacemaker to facilitate a transcatheter or surgical repair of the tricuspid and pulmonary valve as a second step. The Aveir DR leadless pacemaker system was implanted successfully (Figure 1A–1C) using right femoral vein access with ultrasound guidance. The atrial device was placed at the base of the right atrial appendage, the ventricular device at the midventricular septum (AR and VR sensing 1.7 and 6.5 mV; impedance 300 and 570 Ω; thresholds 1.3 V at 0.4 ms and 1.0 V at 0.4 ms, respectively) using the Abbott delivery catheter LSCD201 (sheath size 27F). For both devices, contrast injection was used to confirm the implantation site. Injury pattern and thresholds were tested before and after fixation. A total of 1.5 turns of the radiopaque chevron were performed during fixation under fluoroscopic visualization, following the manufacturer’s recommendations and under fluoroscopic visualization preventing counter-rotation. Postoperatively, no pericardial effusion or groin-related complications were observed. However, on the first postoperative day, the interrogation of the device was challenging owing to repetitive loss of connectivity to the devices. After several attempts, the devices were interrogated successfully, revealing a loss of capture of the atrial device (Figure 2A) and loss of dual-chamber i2i communication. The ventricular device showed satisfactory threshold, sensing, and impedance. A following radiograph of the chest confirmed a dislodgment of the atrial device (Figure 2B). The patient was scheduled for device revision in the electrophysiological laboratory using remote support of Abbott.

At the beginning of the procedure, embolization of the atrial leadless pacemaker into the left renal vein was documented (Figure 1D, Supplemental movie). Again, the right femoral vein was used as the access site with ultrasound guidance. The docking button of the leadless pacemaker pointed toward the inferior vena cava, facilitating a retraction of the pacemaker using the snare of the extraction tool provided by Abbott (Aveir retrieval catheter LSCR111, sheath size 27F) (Figure 1E and 1F). The device was successfully retracted and directly prepared for a new implantation attempt using the Abbott delivery catheter LSCD201. The atrial pacemaker was implanted successfully slightly deeper at the base of the right atrial appendage (sensing 2.4 mV, threshold 1.4 V at 0.4 ms, respectively) (Figure 1G–1I). An interrogation of the device on the following day showed satisfactory thresholds, sensing, and impedance for both devices (Figure 2C and 2D). i2i communication (>80%) was achieved using high i2i communication levels (7 of 7). A femoral artery pseudoaneurysm (diameter 3.6 cm) was successfully treated with thrombin injection. The patient was discharged the next day and remained free of complications during 3 months of follow-up.

## Discussion

Lead dislodgement is a complication in transvenous pacemakers occurring in 1.2%–3.3% of implantations, predominantly owing to mechanical forces.^5^^,^^6^ Atrial lead dislodgement occurs approximately 3 times more often than in ventricular leads.^7^ The reported dislodgment rate of ventricular leadless pacemakers (Aveir) is 0.88%.^8^ However, data on atrial leadless pacemaker dislodgment remain limited. A dislodgment rate of 1.7% has been described for atrial devices, primarily attributed to inadequate helix fixation.^1^^,^^9^

Whether known risk factors for transvenous lead dislodgment—female sex, age, and a higher BMI—apply to leadless pacemakers remains unknown.^5^^,^^10^ In this case, several factors may have contributed to device dislodgment:

• Right atrial dilation owing to severe tricuspid regurgitation likely caused hemodynamic turbulence.
• Previous atrial surgery and ablation, possibly leading to atrial fibrosis, may have reduced mechanical anchoring capacity.
• Severe tricuspid regurgitation might affect optimal anchoring, even in standard implantation sites such as the base of the right atrial appendage.
• Obesity (BMI 40 kg/m^2^) exceeds the cohort average reported in Knops et al^1^ and may have influenced outcomes.

Additional preprocedural imaging—such as computed tomography or transesophageal echocardiography—may help reduce the risk of leadless pacemaker dislodgment in anatomically challenging cases, although this potential benefit remains uncertain. In patients with severe tricuspid regurgitation, lowering right-sided pressures through diuresis before implantation might also decrease dislodgment risk. However, further studies are needed to better understand the underlying risk factors associated with leadless pacemaker dislodgment.

## Conclusion

In this case, dislodgment and embolization of a leadless pacemaker into the left renal vein occurred within 24 hours after implantation. Retraction of the leadless pacemaker and a second implantation attempt of the same device were feasible and successful. High BMI, tricuspid regurgitation, and atrial dilatation may have contributed to the pacemaker dislodgment. The underlying mechanisms and risk factors remain unclear.

## Disclosures

S.R. has no conflicts of interest to declare. J.N. received speaker’s bureau/proctor honoraria from Medtronic and Abbott. R.R.T. received research grants from Medtronic and Biotronik; travel grants from Biosense Webster, Medtronic, Abbott, SentreHEART, and Daiichi Sankyo; and speaker’s bureau/proctor honoraria from Biosense Webster, Medtronic, Abbott, SentreHEART, and Daiichi Sankyo; he is a consultant of Biosense Webster and Biotronik.
