# Supplementary material for: Dislodgment and embolization of an atrial leadless pacemaker
Source: HeartRhythm Case Rep. 2025 Sep 19;11(12):1318–21. doi: 10.1016/j.hrcr.2025.09.009 (PMC12805299; doi:10.1016/j.hrcr.2025.09.009)
Supplement: Supplemental Legend [file mmc2.docx]

**Supplemental data**

**Movie 1:** Angiographic images showing the atrial leadless pacemaker in the left renal vein, snaring and successful retraction of the device and successful re-implantation at the base of the right atrial appendage.
